# Azathioprine, Mercaptopurine, and 5-Aminosalicylic Acid Affect the Growth of IBD-Associated *Campylobacter* Species and Other Enteric Microbes

**DOI:** 10.3389/fmicb.2017.00527

**Published:** 2017-03-29

**Authors:** Fang Liu, Rena Ma, Stephen M. Riordan, Michael C. Grimm, Lu Liu, Yiming Wang, Li Zhang

**Affiliations:** ^1^School of Biotechnology and Biomolecular Sciences, University of New South WalesSydney, NSW, Australia; ^2^Gastrointestinal and Liver Unit, Prince of Wales Hospital, University of New South WalesSydney, NSW, Australia; ^3^St George and Sutherland Clinical School, University of New South WalesSydney, NSW, Australia; ^4^School of Medical Sciences, University of New South WalesSydney, NSW, Australia

**Keywords:** *Campylobacter concisus*, inflammatory bowel disease, immunosuppressive drug, azathioprine, mercaptopurine, anti-inflammatory drug, 5-aminosalicylic acid, enteric bacteria

## Abstract

*Campylobacter concisus* is a bacterium that is associated with inflammatory bowel disease (IBD). Immunosuppressive drugs including azathioprine (AZA) and mercaptopurine (MP), and anti-inflammatory drug such as 5-aminosalicylic acid (5-ASA) are commonly used to treat patients with IBD. This study aimed to examine the effects of AZA, MP, and 5-ASA on the growth of IBD-associated bacterial species and to identify bacterial enzymes involved in immunosuppressive drug metabolism. A total of 15 bacterial strains of five species including 11 *C. concisus* strains, *Bacteroides fragilis, Bacteroides vulgatus, Enterococcus faecalis*, and *Escherichia coli* were examined. The impact of AZA, MP, and 5-ASA on the growth of these bacterial species was examined quantitatively using a plate counting method. The presence of enzymes involved in AZA and MP metabolism in these bacterial species was identified using bioinformatics tools. AZA and MP significantly inhibited the growth of all 11 *C. concisus* strains. *C. concisus* strains were more sensitive to AZA than MP. 5-ASA showed inhibitory effects to some *C. concisus* strains, while it promoted the growth of other *C. concisus* strains. AZA and MP also significantly inhibited the growth of *B. fragilis* and *B. vulgatus*. The growth of *E. coli* was significantly inhibited by 200 μg/ml of AZA as well as 100 and 200 μg/ml of 5-ASA. Bacterial enzymes related to AZA and MP metabolism were found, which varied in different bacterial species. In conclusion, AZA and MP have inhibitory effects to IBD-associated *C. concisus* and other enteric microbes, suggesting an additional therapeutic mechanism of these drugs in the treatment of IBD. The strain dependent differential impact of 5-ASA on the growth of *C. concisus* may also have clinical implication given that in some cases 5-ASA medications were found to cause exacerbations of colitis.

## Introduction

Inflammatory bowel disease (IBD) is a chronic inflammatory disease of the gastrointestinal tract with Crohn's disease (CD) and ulcerative colitis (UC) being the two major forms. The etiology of IBD remains elusive but it is believed that multiple factors such as genetic susceptibility, environmental factor, dysregulated immune response as well as intestinal microbiota, are involved in the development of the disease (Sartor and Mazmanian, 2012). Accumulated evidence supports the role of *Campylobacter concisus* as the initiator of a subgroup of IBD (Zhang et al., 2014a; Zhang, 2015).

*C. concisus* is a Gram-negative spiral shaped motile bacterium (Tanner et al., 1981). Their growth under anaerobic and microaerobic conditions is strongly favored by the presence of H_2_, and also affected by other environmental factors in the gastrointestinal tract such as pH and bile (Lee et al., 2014; Ma et al., 2015). The human oral cavity is the natural reservoir of *C. concisus*. However, it may also colonize the intestinal tract of some individuals and its prevalence in the intestinal tissues has been associated with IBD (Tanner et al., 1981; Lastovica, 2006; Zhang et al., 2009, 2010; Mahendran et al., 2011; Mukhopadhya et al., 2011; Kirk et al., 2016). In addition to IBD, *C. concisus* was frequently isolated from diarrheal stool samples, suggesting its involvement in diarrheal disease (Lindblom et al., 1995; Lastovica, 2006; Kalischuk and Inglis, 2011; Nielsen et al., 2013). Some oral *C. concisus* strains were found to invade human intestinal epithelial cells, further supporting the idea that they can cause enteric disease (Nielsen et al., 2011; Ismail et al., 2012, 2013). Various virulence factors were found in *C. concisus*, such as the zonula occludens toxin (Zot), which was shown to cause prolonged damage to the intestinal epithelial barrier, increase and enhance the response of macrophages to other bacterial species (Mahendran et al., 2013, 2016; Liu et al., 2016).

The thiopurine drugs azathioprine (AZA) and mercaptopurine (MP) are immunosuppressive drugs that are commonly used for induction and maintenance of remission in patients with IBD (Chande et al., 2015, 2016; Timmer et al., 2016). AZA is a prodrug of MP. Once absorbed, AZA is processed to release MP and a glutathionyl derivative of 1-methyl-4-nitroimidazole in the liver or other body cells (Hobara and Watanabe, 1981; Elion, 1993; Gervasio et al., 2000). This process is due to the action of glutathione, which is in part controlled by the enzyme glutathione S-transferase (GST) (Eklund et al., 2006). MP undergoes further enzymatic conversions, leading to the production of the intermediates thioinosine monophosphate (TIMP) and thioxanthine monophosphate (TXMP) and the active metabolite thioguanine nucleotides (TNGs). TNGs act as purine antagonists for DNA polymerase and induce mismatches in DNA synthesis, thus inhibit proliferation of fast-growing cells lacking a purine salvage pathway such as B and T lymphocytes (Nielsen et al., 2001; Sahasranaman et al., 2008).

5-aminosalicylic acid (5-ASA) is an anti-inflammatory drug in the sulfonamide family that is also commonly used to induce and maintain remission in IBD (Hanauer, 2006; Nikfar et al., 2009). The mechanism of action of 5-ASA is believed to be due to the activation of the peroxisome proliferation-activated receptor gamma (PPAR-γ) (Ireland and Jewell, 1990). Activation of PPAR-γ leads to a decreased transcriptional activity of NF-κB and reduction of inflammatory cytokine production (Dubuquoy et al., 2003; Rousseaux et al., 2005).

Currently, limited information is available regarding whether these immunosuppressive and anti-inflammatory drugs used in IBD therapies have an impact on bacterial species in the human gastrointestinal tract. To gain this information, in this study we examined the effects of AZA, MP and 5-ASA on the growth of *C. concisus* and the four other enteric bacterial species. This study found that AZA and MP inhibited the growth of IBD-associated bacterium *C. concisus* and some other enteric bacterial species. AZA showed a greater bacterial inhibitory effect to *C. concisus* than MP. 5-ASA had differential effects on the growth of different *C. concisus* strains. The clinical implications of these findings and the potential therapeutic mechanisms of the bacterial inhibitory effects of the examined drugs were discussed.

## Materials and methods

### Bacterial species included in this study

A total of 15 bacterial strains of five species were used in this study, including 11 *C. concisus* strains and *Bacteroides fragilis* ATCC 25285, *Bacteroides vulgatus* ATCC 8482, *Enterococcus faecalis* ATCC 19433 and *Escherichia coli* K12 (Bachmann, 1972; Gregory et al., 1978; Moschetti et al., 1998). Of the 11 *C. concisus* strains examined, three strains were from patients with CD, four strains from patients with UC, one strain from patient with gastroenteritis and three strains from healthy controls. These 11 *C. concisus* strains were randomly chosen from *C. concisus* strains isolated in our previous studies (Zhang et al., 2009, 2010; Mahendran et al., 2011). *C. concisus* consists of two genomospecies (GS), among the 11 strains included, five strains were from GS1 and six strains were from GS2 (Mahendran et al., 2015; Chung et al., 2016). The other bacterial strains were purchased from the American Type Culture Collection (VA, USA). Detailed information of bacterial species and strains used in this study were provided in Table 1.

**Table 1:** **Bacterial strains used in this study**.

**Strain ID**	**Health status**	**Isolation source**	**Genomospecies**
P2CDO4	CD	Saliva	GS2
P11CDO-S1	CD	Saliva	GS2
P20CDO-S2	CD	Saliva	GS2
P14UCO-S1	UC	Saliva	GS2
P3UCO1	UC	Saliva	GS1
P3UCLW1	UC	Feces	GS1
P3UCB1	UC	Intestinal biopsy	GS1
13826	Gastroenteritis	Feces	GS2
H14O-S1	Healthy	Saliva	GS2
H12O-S1	Healthy	Saliva	GS1
H17O-S1	Healthy	Saliva	GS1
*B. fragilis* ATCC 25285	Appendix abscess	Appendix abscess	
*B. vulgatus* ATCC 8482	NA	Feces	
*E. faecalis* ATCC 19433	NA	NA	
*E. coli* K12	Convalescent diphtheria	Feces	

*GS1, genomospecies 1; GS2, genomospecies 2; The 11 C. concisus strains are underlined; and their GS was examined in our previous studies (Mahendran et al., 2015; Chung et al., 2016). Letters P and H in strain ID indicate strains isolated from patients with inflammatory bowel disease and healthy controls respectively. O indicates oral strains isolated from saliva samples; B indicates a strain isolated from intestinal biopsy; and LW indicates a strain isolated from fecal sample. CD, Crohn's disease; UC, ulcerative colitis; NA, information not available*.

### Effects of AZA, MP, and 5-ASA on *C. concisus* growth

Four concentrations of drugs were examined for their effects on bacterial growth, which were 10, 50, 100, and 200 μg/ml. These concentrations were estimated based on the doses of AZA and MP used in the treatment of IBD. The doses used in the treatment of IBD were 2.5 mg per kilogram of body weight per day for AZA and 1.5 mg per kilogram of body weight per day for MP (Pearson et al., 1995). Given this, the concentrations of AZA and MP entering into the intestinal tract would be 100–200 μg/ml, assuming the body weights are 60–70 kilograms and the volume in the stomach is 1 liter (Sherwood, 2015). The doses of 5-ASA used in the treatment of IBD vary greatly depending on different clinical situations (Wang et al., 2016). The drug concentrations examined in this study were not intended to accurately reflect the concentrations of these drugs in the human intestinal tract, which was impossible to do. All test drugs were purchased from Sigma-Aldrich (Castle Hill, Australia).

*C. concisus* strains were cultured on horse blood agar (HBA) plates under anaerobic conditions supplemented with 5% H_2_ for 48 h at 37°C as previously described (Lee et al., 2014). Bacteria of each *C. concisus* strain were then collected from the plate and diluted to OD_600_ of 0.025 with PBS. An aliquot of bacteria (5 μl) was spread in radial pattern on the agar plates containing different concentrations of AZA, MP or 5-ASA (10, 50, 100, and 200 μg/ml). Plates were then incubated for 48 h. Each treatment was performed in triplicate. Following incubation, bacteria were collected from each plate with 1 ml PBS, from which seven serial dilutions (10^−1^–10^−7^) were prepared. An aliquot (5 μl) of each of the seven dilutions as well as the undiluted original inoculum was then dropped onto HBA plates in quadruplicate. The plates were incubated for 48 h and the colony forming unit (CFU) was determined.

*B. fragilis, B. vulgatus, E. faecalis* and *E. coli* were cultured on HBA plates for 24 h at 37°C under anaerobic conditions supplemented with 5% H_2_ (Whelan and Hale, 1973; Ruoff et al., 1990; Likotrafiti et al., 2016). Following initial cultivation, the effects of AZA, MP and 5-ASA on the growth of *B. fragilis, B. vulgatus, E. faecalis* and *E. coli* were examined under the same cultivation conditions as those used for *C. concisus* except the cultivation period was 24 h instead of 48 h giving the rapid growth of these bacterial species (Whelan and Hale, 1973; Ruoff et al., 1990; Likotrafiti et al., 2016).

The growth of bacterial strains on HBA plates containing different concentrations of AZA, MP, or 5-ASA was expressed as the percentage of the CFU relative to the CFU of the negative control of the same strain. Experiments were repeated three times.

### The controls

AZA and MP were dissolved in dimethyl sulfoxide (DMSO) at a stock concentration of 100 mg/ml. The concentration of DMSO was adjusted to 0.2% for all concentrations of AZA and MP. Bacterial strains cultured on plates containing DMSO at 0.2% were therefore used as the negative controls for AZA and MP. 5-ASA was dissolved in water at a stock concentration of 1 mg/ml. Bacterial strains cultured on plates without any drugs were served as the negative controls for 5-ASA. Tetracycline at final concentration of 0.5 μg/ml was used as antibiotic control for *C. concisus*, with representative strain 13826 being used (Aabenhus et al., 2005).

### Examination of the presence of enzymes required for AZA and MP metabolism in different bacterial species using bioinformatics tools

The enzymes involved in AZA and MP metabolism include GST, HGPRT, inosine monophosphate dehydrogenase (IMPD), GMPS, xanthine oxidase (XO), and TPMT. The presence of these enzymes in the bacterial species examined in this study was identified by searching the bacterial genomes for proteins annotated with the same enzyme names, as well as proteins containing the same enzyme domains. Two protein databases were used including National Center for Biotechnology Information (NCBI) protein database and Universal Protein Resource (UniProt; Apweiler et al., 2004; Pruitt et al., 2007).

Of the 11 *C. concisus* strains included, seven of them have been previously sequenced by our group, the presence of the enzymes were examined in these seven strains as well as the publically available genome of *C. concisus* strain 13826 (accession number CP000792.1; Chung et al., 2016).

### Statistical analysis

Student's *t*–tests (unpaired, two tailed) were used to compare the CFU of bacterial strains in response to AZA, MP and 5-ASA with those of the negative controls. *P* < 0.05 were considered as statistically significant. Statistical analyses were performed using GraphPad Prism version 6 (San Diego, CA). Data were shown as the mean ± SD from triplicates.

## Results

### Effects of AZA, MP, and 5-ASA on the growth of *C. concisus* strains

**Figure 1:**
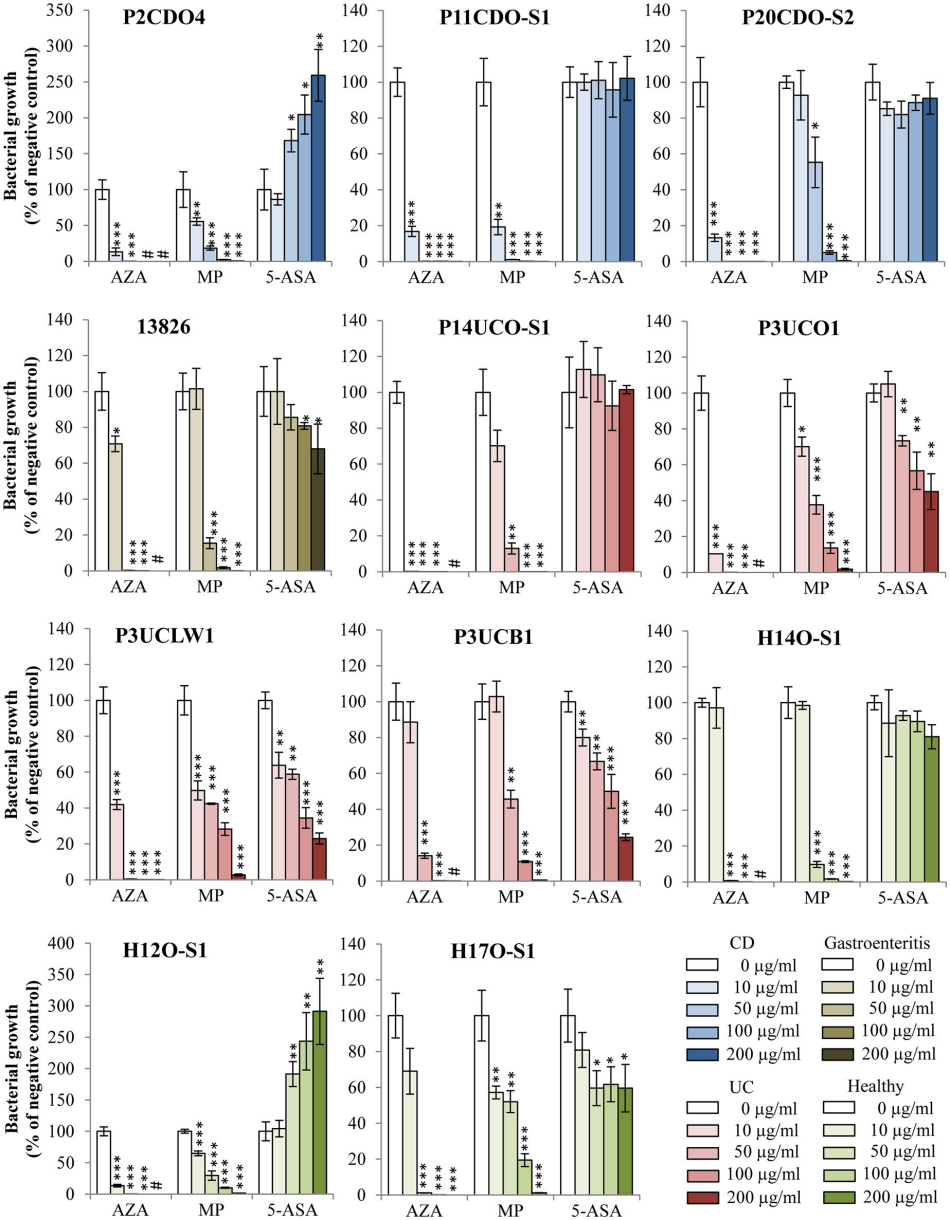
**Effects of AZA, MP, and 5-ASA on the growth of ***C. concisus*** strains**. The growth of *C. concisus* strains on horse blood agar plates containing different concentrations of AZA, MP or 5-ASA was expressed as the percentage of the colony forming unit (CFU) relative to the CFU of their respective negative control. The negative controls were the same *C. concisus* strains grown on HBA plates without drugs. Strains isolated from patients with CD, UC, gastroenteritis and healthy controls are colored in blue, red, brown and green respectively. ^*^Indicates statistically significance (^*^P < 0.05, ^**^P < 0.01, ^***^P < 0.001). ^#^Indicates complete inhibition of growth. CD, Crohn's disease; UC, ulcerative colitis.

AZA inhibited the growth of all 11 *C. concisus* strains examined (Figure 1, Table 2). The CFU of eight strains grown on plates containing 10 μg/ml of AZA were significantly lower as compared to that grown on the negative control plates (*P* < 0.05). On plates containing concentrations of 50, 100, or 200 μg/ml of AZA, the CFU of all 11 strains were significantly reduced as compared to their respective strains grown on HBA plates without drugs (negative controls; *P* < 0.05). *C. concisus* appeared to be very sensitive to AZA. In the presence of 10 μg/ml of AZA, more than half of the strains (6/11, 55%) had over 80% of CFU reduction as compared to their respective negative controls. In the presence of 50 μg/ml of AZA, all strains except for one isolated from intestinal biopsies of a patient with UC (P3UCB1) had over 98% CFU reduction as compared to their negative controls. In the presence of 100 μg/ml of AZA, all strains had more than 99% CFU reduction, and one strain showed complete inhibition of growth in which no colonies were observed. Seven strains showed complete inhibition of growth when 200 μg/ml of AZA was present.

**Table 2:** **Effects of AZA, MP, and 5-ASA on the growth of bacterial strains**.

**Bacterial strain**	**AZA (μg/ml)**				**MP (μg/ml)**				**5-ASA (μg/ml)**			
	**10**	**50**	**100**	**200**	**10**	**50**	**100**	**200**	**10**	**50**	**100**	**200**
P2CDO4	13 ± 5	0.002 ± 0	0	0	56 ± 5	18 ± 3	2.3 ± 0.2	0.49 ± 0.05	86 ± 8	168 ± 16	205 ± 27	259 ± 36
P11CDO-S1	17 ± 3	0.14 ± 0.04	0.23 ± 0.01	0.04 ± 0.01	19 ± 4	1.1 ± 0.1	0.17 ± 0.01	0.005 ± 0.001	100 ± 5	101 ± 10	96 ± 15	102 ± 12
P20CDO-S2	13 ± 2	0.11 ± 0.01	0.003 ± 0.001	0.012 ± 0.002	93 ± 14	55 ± 14	5 ± 1	0.46 ± 0.07	85 ± 4	82 ± 8	892 ± 4	91 ± 9
13826	71 ± 4	0.26 ± 0.02	0.03 ± 0.02	0	101 ± 11	15 ± 3	1.8 ± 0.5	0.06 ± 0.01	100 ± 18	86 ± 7	81 ± 2	68 ± 14
P14UCO-S1	0.17 ± 0.05	0.003 ± 0	0.0008 ± 0.0002	0	70 ± 8	13 ± 3	0.05 ± 0.02	0.0007 ± 0.0001	113 ± 16	110 ± 15	92 ± 14	102 ± 2
P3UCO1	10 ± 0	0.0004 ± 0	5*E*−07 ± 2*E*−07	0	70 ± 5	38 ± 5	14 ± 3	1.8 ± 0.4	105 ± 7	73 ± 3	57 ± 10	45 ± 10
P3UCLW1	42 ± 3	0.37 ± 0.06	0.001 ± 0	0.0002 ± 0	50 ± 5	42 ± 0	28 ± 4	2.7 ± 0.5	64 ± 7	59 ± 3	34 ± 6	23 ± 3
P3UCB1	89 ± 11	14 ± 1	0.22 ± 0.02	0	103 ± 9	46 ± 5	11 ± 0	0.51 ± 0.09	80 ± 5	67 ± 5	50 ± 9	24 ± 2
H14O-S1	97 ± 11	0.69 ± 0.09	0.09 ± 0.01	0	98 ± 2	9.8 ± 1.7	1.7 ± 0.1	0.18 ± 0.02	89 ± 19	93 ± 3	90 ± 6	81 ± 7
H12O-S1	13 ± 2	0.07 ± 0.02	0.0001 ± 0	0	65 ± 4	30 ± 8	10 ± 1	1.7 ± 0.2	104 ± 13	191 ± 20	243 ± 46	291 ± 53
H17O-S1	69 ± 13	1.12 ± 0.09	0.1 ± 0.01	1E-06 + 0	57 ± 4	52 ± 6	19 ± 4	1.1 ± 0.1	81 ± 10	60 ± 10	62 ± 10	60 ± 13
*B. fragilis*	80 ± 6	0.11 ± 0.01	0	0	95 ± 8	25 ± 0	1.3 ± 0.2	0.15 ± 0.01	106 ± 3	95 ± 5	108 ± 11	112 ± 10
*B. vulgatus*	73 ± 6	0.00002 ± 0	0	0	88 ± 12	81 ± 13	65 ± 5	35 ± 4	113 ± 11	121 ± 7	115 ± 20	118 ± 12
*E. faecalis*	101 ± 12	84 ± 10	76 ± 8	79 ± 5	99 ± 11	110 ± 5	106 ± 2	94 ± 10	109 ± 10	100 ± 2	106 ± 12	101 ± 14
*E. coli*	101 ± 10	90 ± 10	77 ± 12	15 ± 2	107 ± 9	106 ± 4	109 ± 20	112 ± 11	90 ± 15	82 ± 12	63 ± 2	48 ± 4

*The growth of 11 C. concisus strains and the other four enteric bacterial strains in the presence of different concentrations of AZA, MP or 5-ASA was expressed as the percentage of the colony forming unit (CFU) in relative to the CFU of the negative control of the same strain. The negative controls were the same bacterial strains grown on horse blood agar plates without drugs. Data presented were triplicates ± SD. C. concisus strains are underlined*.

The growth of the 11 *C. concisus* strains was also inhibited by MP (Figure 1, Table 2). When the *C. concisus* strains were grown on HBA plates containing 10 μg/ml of MP, the growth of six strains was significantly reduced as compared with their respective negative controls (*P* < 0.05). At concentrations of 50, 100, and 200 μg/ml, MP significantly inhibited the growth of all 11 strains as compared with their negative controls (*P* < 0.05). In contrast to what was observed with AZA, MP at concentration of 200 μg/ml did not completely abolish the growth of *C. concisus*; bacterial colonies were still observed in all strains.

The effects of 5-ASA on *C. concisus* growth varied between strains (Figure 1, Table 2). Among the 11 strains examined, the growth of five strains (13826, P3UCO1, P3UCLW1, P3UCB1, and H17O-S1) was significantly inhibited by 5-ASA (*P* < 0.05). In contrast, 5-ASA at concentrations of 50, 100, and 200 μg/ml (*P* < 0.05) significantly enhanced the growth of two *C. concisus* strains, P2CDO4 and H12O-S1. The growth of the remaining four strains did not change significantly in the presence of 5-ASA.

The positive control antibiotic tetracycline had more than 99% of inhibitory effect on the growth of *C. concisus* strain 13826 at concentration of 0.5 μg/ml.

### Effects of AZA, MP, and 5-ASA on the growth of *B. fragilis, B. vulgatus, E. faecalis*, and *E. coli*

**Figure 2:**
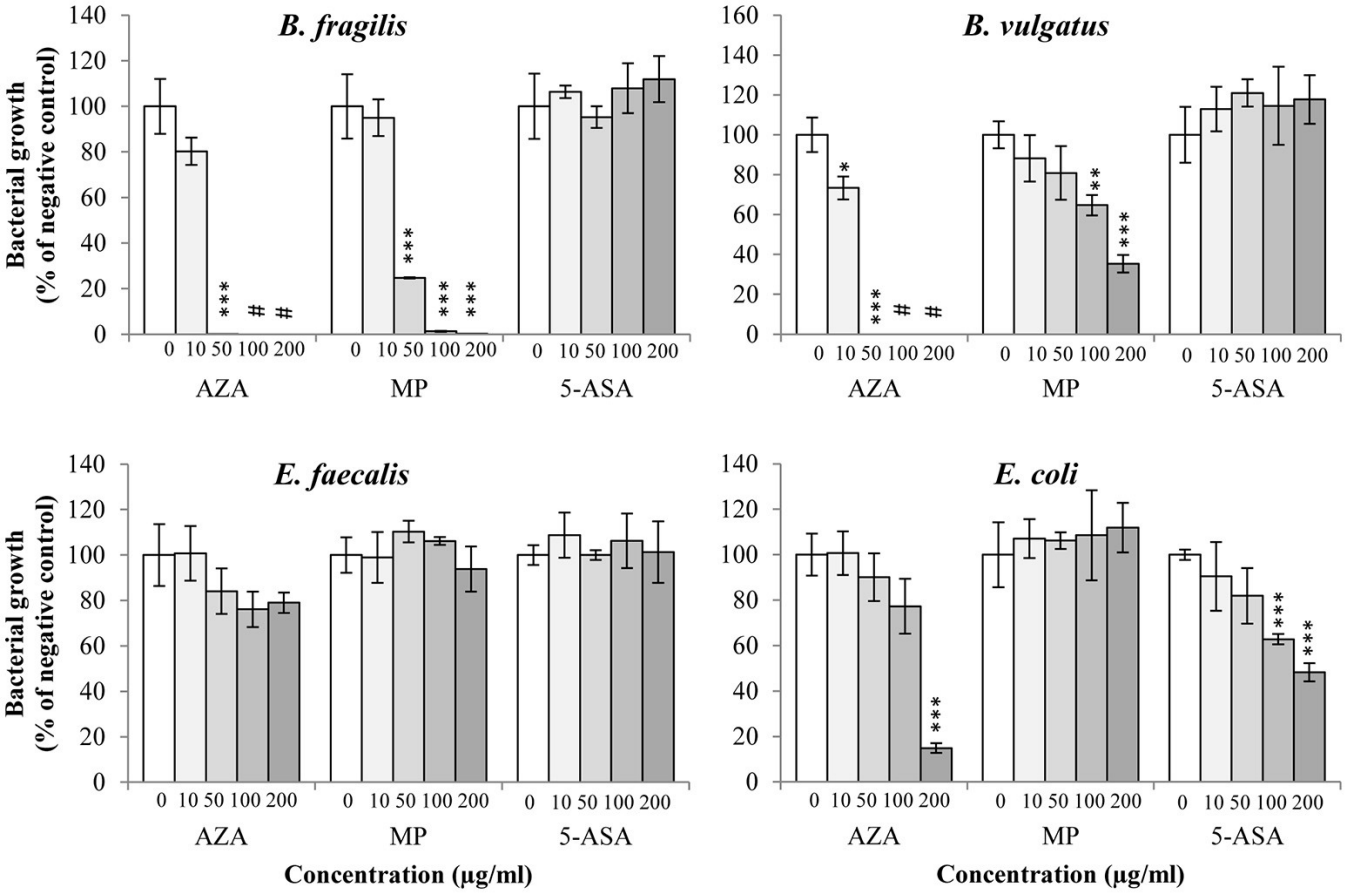
**Effects of AZA, MP, and 5-ASA on the growth of ***B. fragilis***, ***B. vulgatus***, ***E. faecalis***, and ***E. coli*****. The growth of *B. fragilis, B. vulgatus, E. faecalis* and *E. coli* was expressed as the percentage of the colony forming unit (CFU) relative to the CFU of their respective negative control. The negative controls were the same bacterial strains grown on horse blood agar plates without drugs. ^*^Indicates statistically significance (^*^P < 0.05, ^**^P < 0.01, ^***^P < 0.001). ^#^Indicates complete inhibition of growth.

*B. fragilis* and *B. vulgatus* tended to have similar susceptibility toward AZA; their growth was significantly inhibited by AZA at the concentrations of 10 and 50 μg/ml (*P* < 0.05) and completely abolished at higher concentrations (100 and 200 μg/ml; Figure 2, Table 2). The growth of *B. fragilis* and *B. vulgatus* was also significantly inhibited by MP (*P* < 0.05). *B. fragilis* was more sensitive to MP as compared to *B. vulgatus;* a significant reduction of growth of *B. fragilis* was seen in the presence of 50, 100, and 200 μg/ml of MP while a significant reduction of growth of *B. vulgatus* was seen in the presence of 100 and 200 μg/ml of MP (Figure 2, Table 2). 5-ASA did not significantly affect the growth of *B. fragilis* and *B. vulgatus*.

AZA, MP, and 5-ASA did not significantly affect the growth of *E. faecalis*.

The growth of *E. coli* was significantly inhibited by AZA but only at the highest concentration (200 μg/ml; *P* < 0.001), while no inhibitory effect was observed from MP. 5-ASA at 100, and 200 μg/ml significantly reduced the growth of *E. coli* (*P* < 0.001; Figure 2, Table 2).

### Comparison of the bacterial inhibitory effects of AZA and MP

**Figure 3:**
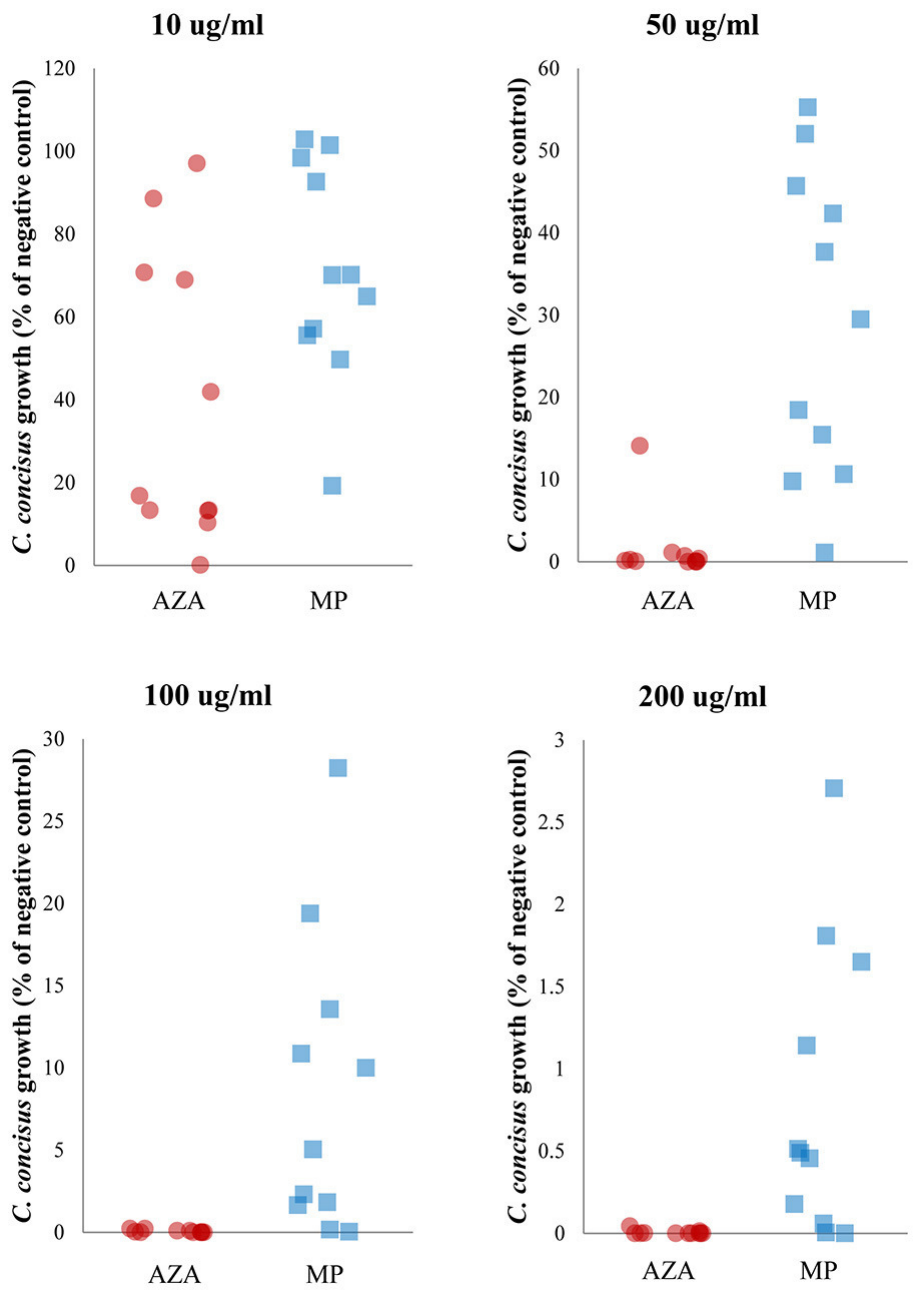
**Comparison of the inhibitory effect of AZA and MP on ***C. concisus*** growth**. *C. concisus* strains were more sensitive to AZA than MP. Six of the 11 *C. concisus* strains (P2CDO4, P20CDO-S2, 13826, P3UCO1, P14UCO-S1, and H12O-S1) exhibited significantly reduced growth in the presence of 10 μg/ml of AZA as compared to that of the same concentration of MP (*P* < 0.05). When AZA and MP were present at higher concentrations (50, 100, and 200 μg/ml), the growth of all 11 *C. concisus* strains were significantly lower in response to AZA than the same concentrations of MP (*P* < 0.01).

The effects of AZA and MP on inhibiting bacterial growth were compared between the growth reduction resulted from the same concentrations of these two drugs and the results were shown in Figure 3, Table 2.

AZA was more effective than MP in inhibiting bacterial growth. Six of the 11 *C. concisus* strains showed significantly lower growth (P2CDO4, P20CDO-S2, 13826, P14UCO-S1, P3UCO1, and H12O-S1) in the presence of 10 μg/ml of AZA as compared with that of the same concentration of MP (*P* < 0.05; Figure 3, Table 2). This trend was more obvious at higher concentrations (50, 100 and 200 μg/ml) in which all 11 *C. concisus* strains in the presence of AZA had significantly lower growth as compared to that of the same strains cultured on the same concentrations of MP (*P* < 0.01; Figure 3, Table 2).

This trend was also seen in other bacterial species including *B. fragilis, B. vulgatus* and *E. coli*. The growth of *B. fragilis* and *B. vulgatus* was significantly lower in the presence of AZA at concentrations of 50, 100, and 200 μg/ml as compared to that of MP at the same concentrations (*P* < 0.001; Table 2). AZA significantly decreased the growth of *E. coli* at concentration of 200 μg/ml, whilst MP did not have significant effects on *E. coli* growth (Table 2).

### Enzymes required for AZA and MP metabolism in the bacterial species

**Figure 4:**
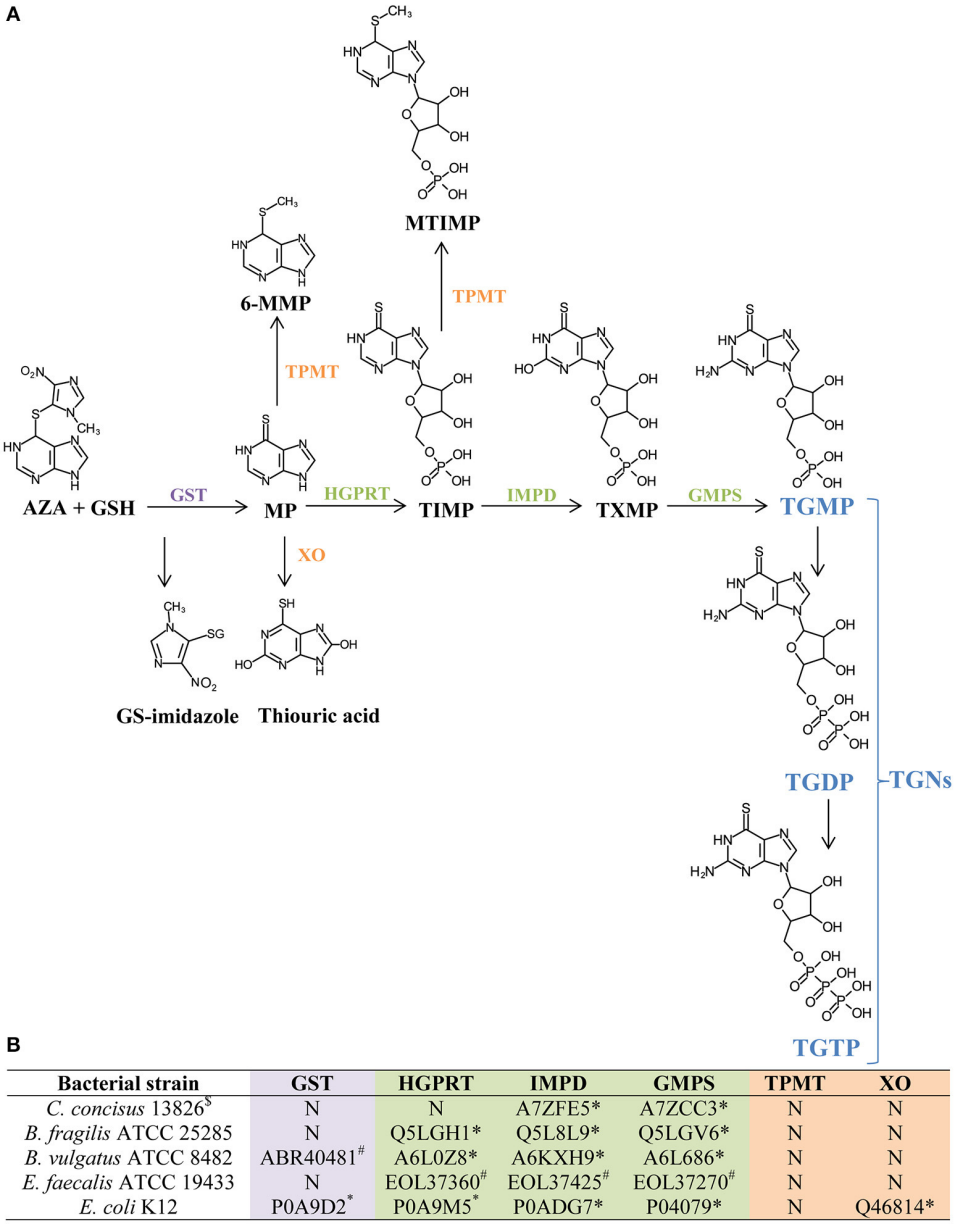
**Identification of enzymes required for AZA and MP metabolism in ***C. concisus*** and other four enteric bacterial species**. **(A)** The process of generating TGNs from AZA and MP. This part of the information was obtained from previous publications (Eklund et al., 2006; Meggitt et al., 2011). TGNs are purine analogs that inhibit DNA synthesis in immune cells which is the therapeutic mechanism of AZA and MP. GSH, glutathione; GS-imidazole, glutathionyl derivative of 1-methyl-4-nitroimidazole; GST, glutathione S-transferase; TPMT, thiopurine methyltransferase; 6-MMP, 6-methylmercaptopurine; HGPRT, hypoxanthine guanine phosphoribosyl transferase; XO, xanthine oxidase; TIMP, thioinosine monophosphate; MTIMP, methylthioinosine monophosphate; IMPD, inosine monophosphate dehydrogenase; TXMP, thioxanthine monophosphate; GMPS, guanosine monophosphate synthetase; TGNs, thioguanine nucleotides; TGMP, thioguanine monophosphate; TGDP, thioguanine diphosphate; TGTP, thioguanine triphosphate. **(B)** The presence of enzymes required for AZA and MP metabolism in *C. concisus* and other bacterial species. This part of the information was obtained in this study by bioinformatics analysis. N, not present. Protein IDs listed are NCBI locus (^#^) or Uniprot protein ID (^*^).

The production of the active metabolites TGNs to inhibit immune cell DNA synthesis by AZA requires a number of enzymes (Figure 4). The presence of these enzymes in the bacterial species and strains included in this study was examined using publically available protein databases annotated from sequenced bacterial genomes. Except for the genome of *E. faecalis* ATCC 19433, the genomes of the four other bacterial species and strains examined were fully sequenced without gaps.

The enzyme GST which facilitates the conversion from AZA to MP, and HGPRT which is required for the formation of the active metabolite TGNs were not found in *C. concisus* strains (Figure 4). In the absence of GST, less MP is produced from AZA; and without HGPRT, conversion of AZA and MP into the active metabolite TGNs is not possible.

GST was not found in *B. fragilis* ATCC 25285 and *E. faecalis* ATCC 19433, however HGPRT, IMPD, and GMPS were found. *B. vulgatus* ATCC 8482 and *E. coli* K12 strains contained all the enzymes required for the metabolisms of AZA to TGNs (Figure 4).

Enzymes TPMT and XO divert the metabolism pathway from TGNs production. TPMT was absent in all bacterial species and strains examined and XO was only found in *E. coli* K12.

## Discussion

In this study, we examined the effects of AZA, MP, and 5-ASA on the growth of IBD associated bacterium *C. concisus* and four other enteric bacterial species. We have also examined the presence of enzymes involving in AZA and MP metabolisms in these bacterial species.

We found that both AZA and MP significantly inhibited the growth of *C. concisus*, a bacterium that was previously found to be associated with IBD (Figure 1, Table 2). The improvement of clinical conditions in IBD patients following the treatment with AZA and MP was believed to be due to the effects of these drugs on reduction of lymphocyte proliferation and proinflammatory cytokines (Derijks et al., 2006). Our finding that AZA and MP have inhibitory effects to *C. concisus* suggests that inhibition or elimination of IBD-causing bacterial species may be an additional therapeutic mechanism contributing to the beneficial effects of AZA and MP in the treatment of IBD. This is supported by previous studies showing AZA and MP also inhibited the growth of *Mycobacterium avium* subsp. *paratuberculosis*, another bacterium that is associated with human CD (Sanderson et al., 1992; Collins et al., 2000; Sieswerda and Bannatyne, 2006; Greenstein et al., 2007; Shin and Collins, 2008).

AZA showed a significantly higher potency in inhibiting the growth of *C. concisus* as compared to the same concentrations of MP (Figure 3). In their use as immunomodulatory medications, AZA is the prodrug of MP. Both AZA and MP are eventually metabolized to purine analogs that interfere with DNA synthesis in immune cells (Nielsen et al., 2001). *C. concisus* does not possess GST, suggesting that the conversion from AZA to MP by *C. concisus* was mainly through the non-enzymatic action. The enzyme GST does not appear to be a critical enzyme in determination of bacterial sensitivity to AZA. For example, GST was not found in *C. concisus, B. fragilis* and *E. faecalis*, however AZA had significant inhibitory effects only to *C. concisus* and *B. fragilis*, not *E. faecalis*. HGPRT enzyme was not found in *C. concisus*, suggesting that most of AZA and MP were not metabolized to purine analogs by *C. concisus*. The more effective inhibitory effects of AZA to *C. concisus* as compared to MP would therefore have to come from AZA itself. This view is further supported by the findings that *E. faecalis* has all enzymes required for converting MP to purine analogs, and that AZA and MP did not significantly affect its growth (Figures 2, 4, Table 2). Nevertheless, the absence or presence of bacterial enzymes in this study was identified using bioinformatics analysis, which requires experimental verification. AZA contains an imidazole ring that is present in many antifungal agents, which may have contributed to the better antibacterial properties of AZA as compared to MP observed in this study (Zhang et al., 2014b).

When taken orally, about 88% AZA and 50% MP are absorbed in the gastrointestinal tract (Cuffari et al., 2000). These absorption rates are affected by both host factors and the mode of drug delivery (Van Os et al., 1996; Tremaine, 1997). Thus, in patients with IBD receiving AZA and MP treatment, the concentrations of these drugs in their gastrointestinal tract vary. Furthermore, *C. concisus* strains have various abilities in resistance to AZA, as judged by the CFU reduction of different strains in the presence of the same concentrations of AZA (Figure 1, Table 2). Clinically, AZA and MP are not effective in all patients and some patients may relapse even under the treatment of AZA and MP. One possible reason contributing to AZA and MP treatment failure may be that the IBD-associated bacterial species such as *C. concisus* have not been successfully inhibited in these patients by these drugs. Future studies should be conducted in patients with IBD to further investigate this issue.

Another interesting finding from this study was that 5-ASA had different effects on the growth of different *C. concisus* strains. While this drug inhibited the growth of some *C. concisus* strains, it increased the growth of the other strains (Figure 1, Table 2). Although 5-ASA is often used in the treatment of IBD, in some cases 5-ASA medications were found to cause exacerbations of colitis (Schwartz et al., 1982). Our finding that 5-ASA promoted the growth of some *C. concisus* strains suggests that the deteriorated clinical conditions in patients with IBD caused by 5-ASA may be due to its effects in increasing the growth of some IBD causing bacterial species in the intestinal tract. In addition to its anti-inflammatory effects through reducing the level of NF-κB, 5-ASA was previously found to inhibit DNA synthesis in human lymphocytes (Elitsur et al., 1989; Ireland and Jewell, 1990). It is possible that 5-ASA has inhibited the growth of some *C. concisus* strains through affecting bacterial DNA synthesis. It is unclear why some *C. concisus* strains showed increased growth in response to 5-ASA. Future studies examining the whole genomes of *C. concisus* strains that had differential responses to 5-ASA may provide further information.

In addition to *C. concisus*, we examined the effects of AZA, MP, and 5-ASA on the growth of another four enteric bacterial species. We found that both AZA and MP inhibited the growth of *B. fragilis* and *B. vulgatus*. *B. fragilis* colonizes the human intestinal tract and accounts for 0.5% of the commensal human colonic flora, but also causes various human infections (Wexler, 2007). The enterotoxigenic *B. fragilis* strain has been implicated in IBD (Prindiville et al., 2000). *B. vulgatus* and *E. faecalis* were shown to induce colitis in animal models of IBD (Onderdonk et al., 1981; Balish and Warner, 2002). Our findings that AZA and MP have inhibitory effects toward *C. concisus* and other IBD-associated bacterial species suggest that it may be possible to develop non-absorbable forms of AZA or MP to inhibit IBD-associated *Campylobacter* species and *Bacteroides* species in the gastrointestinal tract, which can be used as a strategy for long-term IBD treatment with reduced toxicity to the hosts. Low concentration of AZA (10 ug/ml) showed a much greater inhibition to *C. concisus* than other bacterial species, suggesting the possibility of using low concentration of non-absorbable forms of AZA to treat a subgroup of IBD initiated by *C. concisus* without disturbing the balance of microbiota of the gastrointestinal tract.

The findings from this study and previous studies that AZA, MP, and 5-ASA had different effects on the growth of multiple bacterial species residing the human gastrointestinal tract suggest that these drugs may influence gut microbiota in general. The changed microbiota caused by these drugs may further affect the mucosal immunity or colonization of opportunistic pathogens in the gastrointestinal tract of patients with IBD.

The data presented in this study were from laboratory culture of individual bacterial species. Despite their strong potential clinical implications, direct examination of the effects of these immunosuppressive and anti-inflammatory drugs on the growth of bacterial species in the human gastrointestinal tract should be conducted using saliva, biopsy and fecal samples collected from patients with IBD prior to and following treatment, which will provide information regarding the effects of these drugs on the bacterial species community in the human gastrointestinal tract.

## Conclusions

In summary, this study found that clinically used immunomodulating drugs AZA and MP in the treatment of IBD inhibited the growth of IBD-associated *Campylobacter* species *C. concisus*, suggesting an additional therapeutic mechanism of these medications in treatment of IBD. The anti-inflammatory drug 5-ASA may inhibit or promote the growth of *C. concisus*, depending on the properties of the strains, whether this is related to 5-ASA induced deteriorating clinical conditions occurring in some patients with IBD requires further investigation. Furthermore, this study found that AZA, MP, and 5-ASA also inhibit the growth of other enteric bacterial species such as *B. fragilis, B. vulgatus*, and *E. coli*; however, higher concentrations of these drugs are required.

## Availability of data and materials

The dataset supporting the conclusions of this article is included within the article.

## Author contributions

FL, RM, and YW performed the experiment. LL provided important feedback on pharmaceutical aspect. LZ, SR, MG, and FL conceived the project. FL, LZ, RM, SR, MG, LL, and YW wrote the manuscript. All authors have approved the final version of the manuscript.

## Funding

This work is supported by a Faculty Research Grant awarded to LZ from the University of New South Wales (Grant No: PS35329).

### Conflict of interest statement

The authors declare that the research was conducted in the absence of any commercial or financial relationships that could be construed as a potential conflict of interest.

## Abbreviations

5-ASA: 5-aminosalicylic acid
6-MMP: 6-methylmercaptopurine
MP: mercaptopurine
AZA: azathioprine
CD: Crohn's disease
GMPS: guanosine monophosphate synthetase
GSH: glutathione
GST: glutathione S-transferase
HGPRT: hypoxanthine guanine phosphoribosyltransferase
IBD: inflammatory bowel disease
IMPD: inosine monophosphate dehydrogenase
MTIMP: methylthioinosine monophosphate
TGDP: thioguanine diphosphate
TGMP: thioguanine monophosphate
TGNs: thioguanine nucleotides
TGTP: thioguanine triphosphate
TIMP: thioinosine monophosphate
TPMT: thiopurine methyltransferase
TXMP: thioxanthine monophosphate
UC: ulcerative colitis
XO: xanthine oxidase
XDH: xanthine dehydrogenase.
